# Scientists’ Understandings of Risk of Nanomaterials: Disciplinary Culture Through the Ethnographic Lens

**DOI:** 10.1007/s11569-017-0297-2

**Published:** 2017-08-03

**Authors:** Mikael Johansson, Åsa Boholm

**Affiliations:** 1 Gothenburg Research Institute, University of Gothenburg, Box 100, 405 30 Gothenburg, Sweden; 2 School of Global Studies, University of Gothenburg, Box 100, 405 30 Gothenburg, Sweden

**Keywords:** Nanotechnology, Scientific experts, Risk, Ethnography

## Abstract

There is a growing literature on how scientific experts understand risk of technology related to their disciplinary field. Previous research shows that experts have different understandings and perspectives depending on disciplinary culture, organizational affiliation, and how they more broadly look upon their role in society. From a practice-based perspective on risk management as a bottom-up activity embedded in work place routines and everyday interactions, we look, through an ethnographic lens, at the laboratory life of nanoscientists. In the USA and Sweden, two categories of nanoscientists have been studied: upstream scientists who are mainly electrical and physical engineers and downstream scientists who are toxicologists, often with a more multidisciplinary background, including physics, chemistry, biology, and engineering. The results show that although the two groups of scientists share the same norms of appropriate laboratory conduct to promote safety and good science practice, they have very different perspectives on risk with nanomaterials. Upstream scientists downplay risk; they emphasize the innovative potential of the new materials to which they express an affectionate and personalized stance. The downstream scientists, instead, focus on the uncertainties and unpredictability of nanomaterials and they see some materials as potentially highly dangerous. The results highlight the ambiguous and complex role of scientific experts in policy processes about the risk and regulation of nanotechnology.

## Introduction

Nanomaterials are compounds manipulated at the nanometer scale, i.e., the level of single atoms and molecules. More and more consumer products, such as cleaning products, clothing, and personal care products, are manufactured from engineered nanomaterials at the same time as the hazard potential for humans and the environment is largely unknown [1]. When bulk materials are separated into nanoparticles, the surface-to-volume ratio increases, making nanosized materials more chemically reactive. The smaller size of the particles can also influence toxicity ([2]: 157–158; [3]: 485). Nanoparticles can pass through cell walls and accumulate inside the body. Inhaled nanoparticles, for example, can enter the bloodstream and be transported to organs where they accumulate and possibly create adverse health effects. While the European Union (EU) adopts a more process-oriented stance towards regulating nanoparticles, the USA is more product-oriented [4]. Process-oriented risk assessment focuses on nanomaterials in themselves as a potential threat, while product-oriented risk assessment targets products made from nanomaterials. The EU has regulated nanomaterials in cosmetics and defined some types of nanomaterials in electronic equipment as hazardous substances [5]. Meanwhile, in the USA, nanosized particles are not regulated differently from the bulk form, leading to no special regulations ([6]: 87–88). Regulation of (engineered) nanomaterials is fragmented in the EU as well as in the USA due to a variety of existing bodies of legislation in a number of policy areas such as environmental protection, chemicals, food safety, drugs, and work place safety [7, 8]. The challenges for risk assessment and risk management of nanomaterials are substantial [9] due to high uncertainty regarding potential negative effects and their probability, lack of information regarding actual commercial use and exposure, difficulties to implement and enforce regulatory regimes, and lack of standardization of toxicological assessment.

In the extensive literature on the relationship between science and society, scientific experts are generally agreed to have a key role as agenda setters for societal risk issues [10, 11]. Their expert knowledge and understanding of a field, and in particular of potential gains and harmful outcomes of applications of science and technology, influence policy development, regulation, innovation, and media representation. Scientists’ perspectives also contribute to shape public understanding, especially in fields such as nanotechnology where the public has little knowledge and lack well-formed opinions [12]. Not surprisingly, there is a substantial body of research that has explored how scientific experts understand risk in relation to a science field, how they understand benefits, and how they look upon issues of regulation.

One strand of research has explored cultural dimensions relating to value systems and philosophical assumptions, epistemologies, and worldviews that influence scientists’ perception of risk [13–16]. Perceived risks by scientists are explained by factors such as disciplinary background, organizational affiliation, work experience, and worldview. Barke and Jenkins-Smith [14] describe how scientists operating in industry de-emphasize technological risk compared to scientists employed by universities. Specifically regarding nanotechnology, Powell ([16]:175) note that “upstream” scientists—engineers, chemists, physicists, and material scientists—who develop new nanomaterials, and “downstream” scientists—toxicologists, epidemiologists, and public health scientist—who investigate environmental and health effects of the new technologies. While upstream scientists generally perceive little or no risk associated with the new technologies, downstream scientists underscore that there are indeed potential new risks [16]. Bertoldo et al. [15] had similar findings in a study of scientific experts in the field of nanotechnology. Upstream scientists saw nanotechnology as presenting opportunities, not risks, while other types of scientists identified both benefits and risks with the new technology.

The literature on scientific experts’ understanding of science and technology within a societal context and how they perceive risk and their attitudes to regulation in most cases draw survey and interview data. Less studied is how their understandings of risk are embedded in work practices and organizational behavior. To manipulate or investigate nanomaterials, scientists surround themselves with all kinds of technology. In fact, they are enmeshed in a human-made techno-scientific landscape comprising a wide variety of machines and gadgets, ranging from computers to Bunsen burners and Petri dishes. The laboratory environment in itself serves as creator and mediator of meaning (see [17–20]). This paper draws inspiration from ethnographic perspectives on scientific environments in general and from the laboratory life of nanoscience in particular [21–28].

In social sciences risk research, there is a growing interest in the practical organizational dimensions of risk governance, risk management, and safety work ([29–34]. A focus on practice in risk studies provides a “bottom up” perspective exploring how risk is practically embedded and constituted from everyday routines within social contexts ([33, 34]: 3–9). From a practice-based perspective on risk perception, this paper contributes to knowledge of how scientific experts view risks in relation to nanomaterials. The aim is to look at how downstream and upstream nanoscientists perceive risk [16] and how their understanding influence and are influenced by laboratory routines and protocols. Through an ethnographic lens, we focus on laboratory work practice and understandings of risk of two categories of scientific experts (USA and Sweden) working with nanomaterials. We place scientist’ understanding of risk of nanomaterials in a context of laboratory work practice.

Previous research in psychological studies of risk perception have explained how individual preferences to accept risk derive from subjective judgements formed by cognitive heuristics that people use to sort and assess complex information [62]. Such psychometric risk perception studies [35, 36, 63, Slovic et al. 1980) showed that lay people and experts perceived risk differently. One explanation for this difference in perceived risk was a “deficit model” postulating that lay people have inadequate knowledge of risk. It was argued that lay people perceive risk by the use of heuristics and not (as experts do) from assessment of facts and statistical probability. Lay people therefore overemphasize some risks or underestimate others (Sunstein 2002). However, psychometric studies of risk perception comparing experts and the public have been criticized for methodological flaws [37, 38]. Arguably, there is little empirical support for the idea that experts judge risk differently from non-scientists. Expert as well as public opinion on risk is shaped by a diverse range of personal and professional factors including semantic frames for organizing information into meaningful patterns and structures [16]. For example, a study by Thomas et al. [39] shows that expert judgements of probability estimates (regarding sea level rise in a climate change scenario) depend on heuristics, choices about what information and methods to use, and personal dispositions towards optimism or pessimism in looking into an uncertain future.

Experts have been shown to assess information on risk differently, even within the same field of expertise. Why is this so? One explanation put forward is that experts’ disciplinary background influences how they perceive risk and how they understand risk conceptually [13]. Their discipline promotes certain epistemological and philosophical perspectives above others. Diverging viewpoints among experts, as to how they perceive and assess risk, has also been explained by organizational role. How do experts understand their professional responsibility: is it to warn the public or vulnerable groups of potential hazards, or is it to reassure members of the public that they should not overly worry about potential hazards [40]? Their domain of expertise and their expert role in society matter [41]. The organizational affiliation of experts, whether they are employed by industry, government, or academia, has been shown to influence how they perceive risk within their field of expertise. Barke and Smith [14] found that scientific experts at universities had a stronger tendency to rate risks of nuclear energy and nuclear waste as bigger, than experts working in more direct relationship with the nuclear industry. A study by Murphy [42] shows that organizational affiliation, whether experts were employed at agencies, industry, or independent research institutions, shaped their understanding of risk related to tobacco smoking. Perceived risk among scientists varies due to disciplinary background, work experience, and worldview.

There are a number of studies that focus explicitly on risk perception and views on regulation by nanoscience experts. In a study using the psychometric paradigm, lay people’s and experts’ perceptions of risk with nanotechnology were compared [43]. For both groups, perceived dread and trust in government agencies explained perceived risk. Experts had higher trust in government agencies. From another theoretical perspective Powell [16] compared two groups of scientists and their ways of using narratives of uncertainty and risk to create broader understandings of nanotechnology. A main finding was that “upstream” scientists (who directly work with developing nanomaterials) downplay risk, while “downstream” scientists (who study the effects of nanomaterials) accentuate risk. Powell explains this finding by arguing that different scientists (depending on disciplinary orientation) assume different roles in society, that is to say, whether they see themselves as vehicles of technological innovation and progress or as advocates of public and environmental health and safety.

A study by Besley et al. [44] of a group of American nanoscientists identified two major concerns over risk. The experts identified health and environmental risks on the one hand, “social risks” (threats to privacy, weapons, and economic bad outcomes) on the other. In a study of factors that scientific experts thought would influence public opinion of nanotechnology, Gupta et. al [12] found that the area of application was crucial. The factors identified, understood by the experts to influence public opinion, were the extent to which the application was seen as beneficial, useful, necessary, real, and whether the user is in physical proximity with the device or application.

Corley et al. [45] has investigated scientific expert’s opinions about regulation of nanotechnology. They found that support for regulation was positively correlated with risk perception; not surprisingly, higher level of perceived risk was associated with higher support for regulation. They also noted a gender effect; female scientists were in general more supportive of regulation. Gupta et. al [46] investigated perceptions of risk and benefits with nanotechnology in relation to 15 areas of application. They found that for both experts and lay people, acceptance of nanotechnology is explained by perceived benefit and usefulness and whether the application is judged to be necessary. In addition, experts identified the extent to which end users come into direct physical contact with the application as crucial to societal acceptance. Acceptability of nanotechnology due to scientists’ disciplinary orientation was investigated by Chenel et al. [47] within the application area of nanomedicine. The sample included two categories of disciplines (“disciplinary cultures”): natural sciences and engineering, and social sciences and humanities. Differences were found between the two categories of disciplinary culture, especially regarding how specific innovations of nanomedicine were construed and believed to be acceptable. In a Dutch study by van Dijk et al. [48], the attitudes of expert stakeholders were studied with regard to perceived risk and benefit of some applications of nanotechnology. Perceived risk and benefit explained attitude. The experts were more positive about applications in medicine and less positive about applications in the food sector. Attitudes to applications of nanotechnology in the food and medicine areas were explained by other factors such as urgency, uncertainty, and concern over negative public responses. A conclusion of this study was that experts’ attitudes to nanotechnology innovation are explained by a complex set of factors where risk and benefits play a part but are not exclusive dimensions.

A study by Kim et al. [49] has investigated expert scientists’ views on regulation of nanotechnology. They found that opinions varied considerably between the experts scientists. Three clusters of science roles vis-à-vis regulation were identified: “cautious innovators” (favoring local regulation and public involvement), “nanoregulators” (emphasizing top down regulation emanating from national and international levels), and “technology optimists” (who were skeptical about regulation, and who thought that nanotechnology should be allowed to fast advance without interference). In another American study of scientists’ views on regulation of nanotechnology, Corley et al. [50] found some disciplinary differences: chemists were less in favor of regulation than biologists. Risk perception explained attitudes to regulation. Perceptions of benefits were not significantly related to norms regarding regulation. The areas of application understood to be most in need of regulation were bioengineering/human enhancement, medicine, synthetic biology, and cosmetics. Yet, another American study by Beaudrie et al. [51] investigated how nanoscientists and engineers understood the preparedness of government agencies to regulate nanotechnology. The sample consisted of three subgroups: nanoscientists and engineers, environmental health and safety scientists, and regulatory decision-makers and scientists. While all three groups shared the opinion that regulatory agencies were unprepared to regulate nanotechnology, there were distinct differences between the groups. The regulatory decision-makers/scientists had much less confidence in agency preparedness. Confidence in regulatory preparedness was explained by perception of risks as novel, uncertain, and difficult to assess. Trust in regulatory agencies, views about responsibilities of stakeholders, and broader socio-political values also had some explanatory power.

## Method

Ethnography entails the systematic study of people and cultures, and in anthropology, it has become synonymous with the written products of long-term participatory observations of everyday life practices in settings were people gather, interact, and work (see [52]: 295–302). Ethnographic fieldwork can encompass in principle all kinds of contemporary social and cultural contexts, from rituals among indigenous peoples to meetings among city planners. The aim is the same, to understand a society or group from the inside out, to engage as an outsider with people in their daily life to grasp their outlook of the world. In order to study a specific setting, like for example nanoscientists at work, the anthropologists need no specific education in the natural science fields which constitute nanoscientific research. Lack of skill in nanoscience can actually be an advantage. People under study are often more relaxed if they perceive the ethnographer as an outsider, who does not have any stake in what they do and engage in as part of life. To feel oneself being evaluated or assessed by the researcher can create tensions and contribute to unwillingness to be open and reflective. Being part of a scientific environment without proper belonging makes it easier to understand practices and understandings from an outside perspective.

This study covers both upstream and downstream nanoscientists. Upstream and downstream nanoscientists have been shown in previous research to differ in risk perception due to disciplinary culture [16, 47]. The sample of upstream scientists includes electrical and physical engineers and the downstream sample includes toxicologists who often have a more multidisciplinary background, including physics, chemistry, biology, and engineering. Work practices, routines, and laboratory environments are clearly part of a disciplinary “outlook on the world,” fostering practical everyday experiences of nanomaterials as potential risks.

The empirical material for the study, assembled by the first author, relies on participatory observation in laboratory environments where scientists conduct their work, complemented by semi-structured interviews using open-ended topical questions that allow the interviewees to elaborate freely (see [53]). The semi-structured interviews were accompanied with participatory observation, for example, by attending lunches and seminars, following scientists in the laboratory, chatting in the coffee room, and occasionally going out and having a beer with a scientist. In total, the first author spent 2 years of fieldwork at two universities, 1 year in Sweden, and 1 year in the USA. At the first field site in Sweden, interviews were conducted with 51 nanoscientists and at the second field site in the USA, interviews were made with 23 nanoscientists and 29 toxicologists.

As stated above, this paper is based on fieldwork at two laboratory sites situated at universities on two continents. The first field site was at the Department of Microtechnology and Nanoscience (MC2), Chalmers Technical University, in Gothenburg, Sweden (2003–2004), and the second field site was at the University of California, Santa Barbara (UCSB) and University of California, Los Angeles (UCLA), in the USA (2009).

## Setting the Scene

The MC2 building at Chalmers Technical University, in Gothenburg, Sweden, came into place during the 1980s microtechnology boom. In those days, microtechnology was popularized much as nanotechnology was in 2003–2004. Chalmers University wanted to gather all its microtechnology research under one roof, and a new building was constructed to house it. The building is located on a rocky slope beside the physics department, not for convenience but out of necessity, as this was the only land available at the time. The building was finished in June 2000 and by then microtechnology had become nanoscience, as research had shifted from the micrometer to nanometer scale. At the time of the fieldwork, MC2 was described as a world-class facility [54].

Although the building was finished in 2000, it was not until 2003, when the first author started his fieldwork, that MC2 became an independent department at the university. At that time, approximately 200 researchers from around the world were conducting nanoresearch at MC2. On the bottom floor, we find the heart of the facility, the cleanroom laboratory. Without a cleanroom, research at the nanoscale would not be possible. When experimenting with miniscule particles at the nanometer scale, the experiments must be protected from the surroundings, as vibration, dirt, light, and other pollutants can destroy the particles. This requires a laboratory that filters out all kinds of contaminating factors. In this laboratory surrounding, humans must control and cover themselves so as not to be a source of pollution. Although not all scientists at the MC2 facility use the cleanroom, all experiments at one stage or another need the cleanroom facility. In this sense, the cleanroom is a crucial marker of nanoscience.

**Fig. 1:**
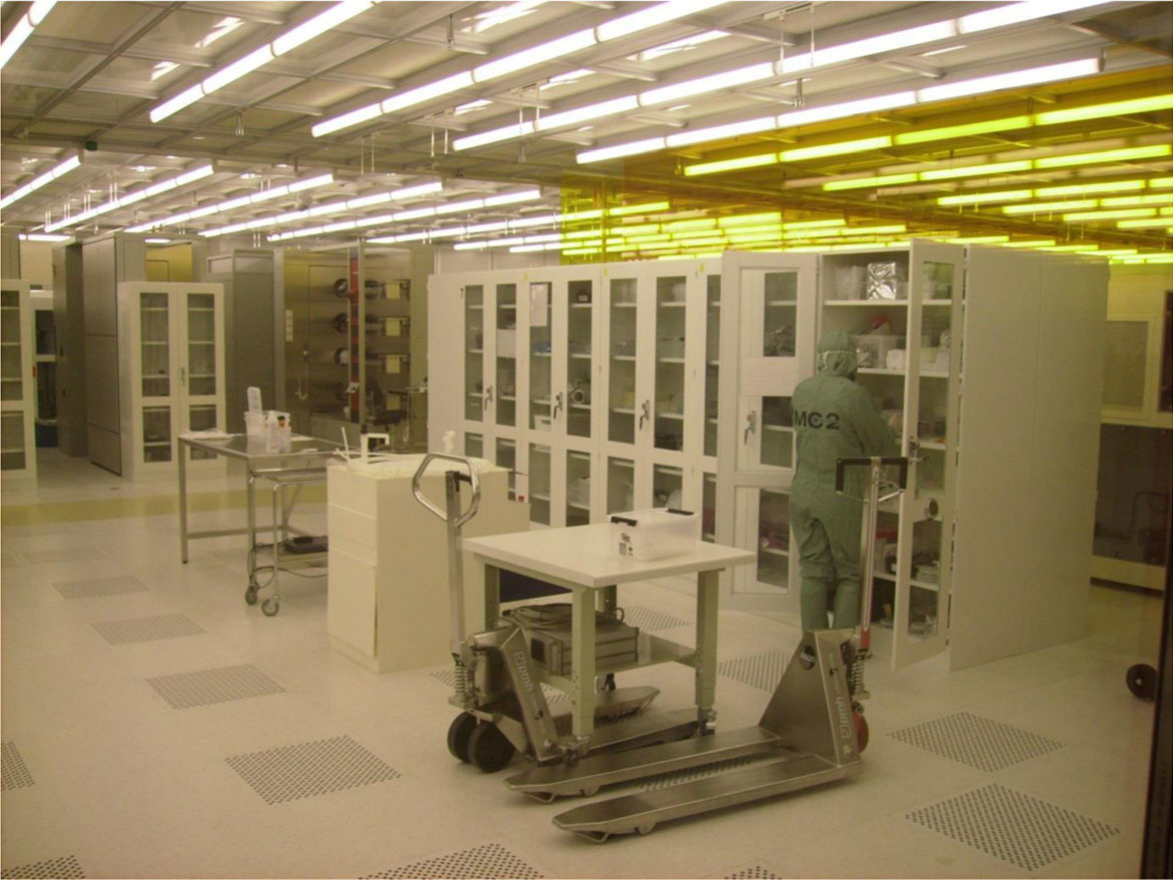
Inside the cleanroom (photo by main author)

In 2008, the first author finished his dissertation on the nanoscientists at the MC2 facility [22] and obtained a postdoctoral position for 2 years at the University of California in Santa Barbara (UCSB). By this time, nanoscience had become more or less ordinary science and Santa Barbara had two cleanroom facilities, one for training and a larger one for experiments. Compared to the stricter cleanroom regime at MC2, the conduct at UCSB was more relaxed, with people chatting and sitting around in chairs. While the MC2 cleanroom was mostly for scientists, the UCSB cleanroom also allowed private companies, who could buy laboratory time at the facility. Many of the PhD students and postdoctoral researcher sat UCSB worked part time in various companies to support their scholarly work. The nanoscience environment at UCSB was less exclusive than at the MC2 facility, and people who considered themselves nanoscientists came from several research groups around the campus. The cleanroom had a total of over 500 registered users, including people from both the university and private corporations (Fig. 1).

During his stay in Santa Barbara, the first author was allowed to conduct participatory observation of toxicologists working at the University of California’s Center for Environmental Implications of Nanotechnology (UC CEIN). This center was multidisciplinary and included several universities as of 2009. Fieldwork was made at two sites, UCSB and UCLA. Although the number of employees fluctuated, around 50–60 people were working at UC CEIN at the time of fieldwork. Many of the scientists were trying to determine whether nanoparticles were hazardous to marine life. As the toxicologists were multidisciplinary, they used various small laboratories suited to their experiments, conducted either alone or with a few colleagues.

**Fig. 2:**
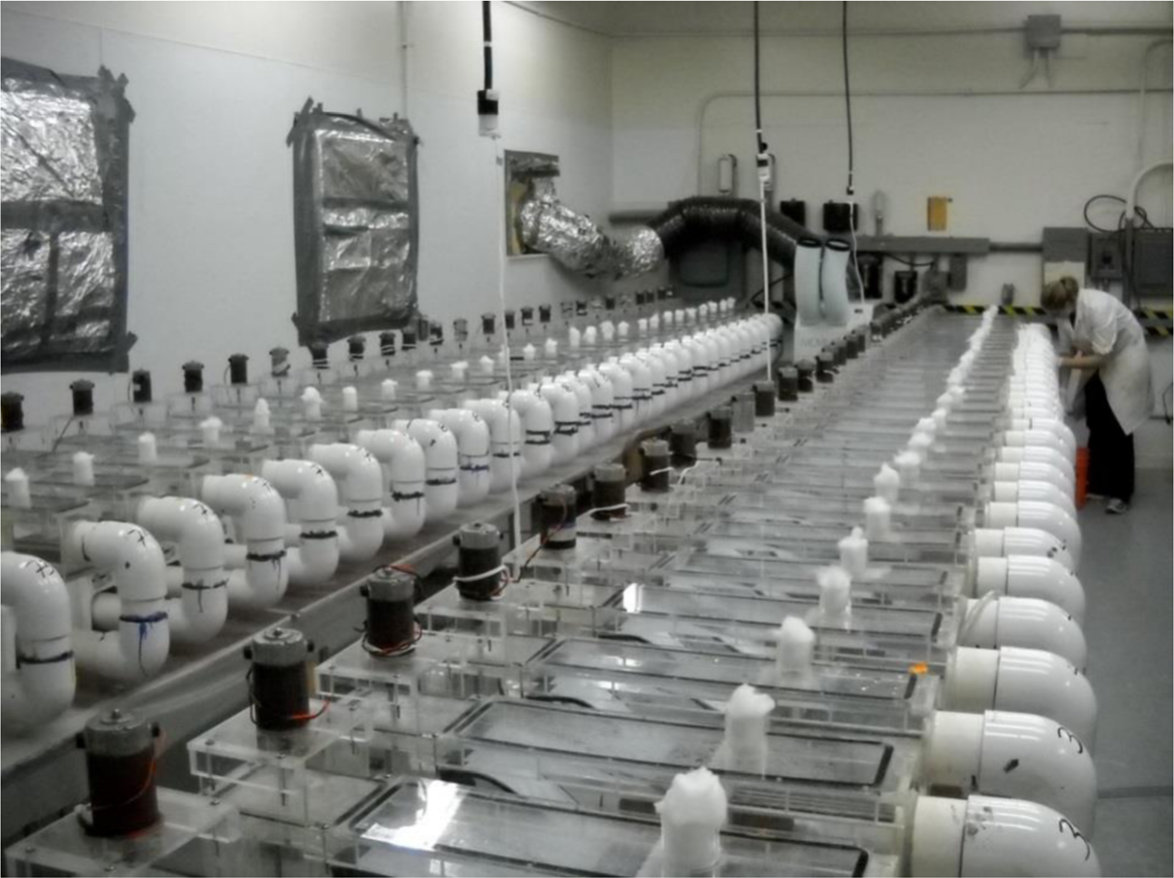
Water tanks used for a nanotoxicology experiment (photo by main author)

One of the more visually interesting toxicology laboratories housed 100 water tanks that were used to investigate whether nanoparticles were hazardous to snails (Fig. 2). Before each experiment, the tanks needed to be thoroughly sterilized. A huge problem was unwanted algae growth in the tanks, so even the rocks used in the experiments needed to be sterilized. As snails in nature are full of contamination and disease, the laboratory had its own snail farm to supply snails for experiments, each of which used 500–1000 snails. When the tanks were all cleaned, the snails were put into the tanks and exposed to various types of nanoparticles at different dosage levels. After 2 months, the experiment was finished and the snails were ground up and examined for nanoparticles. At the time of the fieldwork, there were plans at the laboratory to expand the facility to also include experiments of fish but, as one the scientists said, “the more complex the system, the harder the experiment”.

## Protocols as Modes of Risk Management

Both upstream and downstream nanoscientists follow protocols in their laboratory work practice. In laboratory landscapes, protocols are at the core of research activity. Protocols are understood to condition “good” science, and violation of protocol indicates poor science. Protocols have a strong normative effect and are integral to laboratory work. The ethnographic fieldwork shows that protocols are followed not because of beliefs about risk or ideals about risk management, but because protocols are integral elements of the laboratory scientific landscape. The two groups of nanoscientists both follow the same type of protocols, for the same reasons. In a broad sense, protocols are norms and regulations that nanoscientists need to follow to be safe and to conduct good science. Both upstream and downstream nanoscientists emphasized that science is done by following protocols. Protocols constitute a crucial element of the laboratory landscape, where they ease anxiety about potential hazards.

Since different compounds, when going down in size, get new properties on the nanometer scale, it is not at all clear how protocols designed for risk management of larger scale compounds are actually useful protection aids against nanoparticles. The scientists are aware that there are uncertainties associated with nanomaterials but that there is no other solution than to follow procedures designed for larger materials, often formulated for micrometer-level experiments. One nanoscientist said:

We can’t live in fear—scientists don’t have much room for fear. It’s not that we drink them [i.e. nanoparticles]. We have protocols and we follow them. When it comes to nanoparticles, we don’t know. We try to figure out what is reasonable and limit exposure. We are conscious but not afraid.

Both upstream and downs stream nanoscientists who work in large laboratories told that they oversee their colleagues to be sure that they follow protocol. Protocols are an essential part of the routines of working life in a laboratory environment. Following protocol is done through routine behavior to the effect that the scientists seem unaware that they are doing so. When the first author was on the mailing list for the large laboratory in Santa Barbara, he received weekly e-mails that dealt with violations of protocol.

Protocols can in practice often be contradictory, ambiguous, or vague. For example, one area of concern related to the demarcation between office space and laboratory space, which might be problematic in a shared environment. It happened that the office space with computers was in the same area as the actual lab. The protocol stated that lab coats were needed when working in the lab, but that lab coats to prevent contamination, needed to come off in the office area. It was therefore part of the protocol in this particular laboratory environment, to take off the lab coat each time one entered the office space. The border between office space and laboratory space was however not clearly demarcated and invisible to outsiders.

Another example relates to protective gear. Once a scientist in a lab needed to use a particular machine to measure the level of metal nanoparticles in samples, the safety protocol for handling test tubes required that lab gloves be worn. At the same time, the safety protocol for using the machine’s keyboard ruled out gloves, as residues from gloves could contaminate the keyboard. These two conflicting protocols implied that each time the toxicologist needed to change a sample, he or she needed to put on a new pair of gloves, which was not really practical when there were dozens of samples to be tested. The solution for dealing with these two conflicting protocols was a procedure that the toxicologists called “the Michael Jackson,” in which the toxicologist kept the glove on the left hand for manipulating samples while the right unprotected hand was used to type on the keyboard. As both hands were needed to change the samples in the machine, “the Michael Jackson” inevitably resulted in increased risk of exposure to nanomaterials, a fact not considered by the toxicologist.

In most single-person laboratories observed, there are just a few posted signs regulating safety practices. In a way, a private laboratory can be seen as a private space, like a home: people do not post lots of safety signs in their own homes, as they trust their own judgment and, in any case, nobody is around to detect a violation of protocol. Often, the scientist who uses such a lab works without protective gear, believing that “I know what I am doing,” and may even discard some protocols due to trust in personal judgment. One example is about refrigerators: most labs have refrigerators used for storing samples. As refrigerators also are used to store food, there is a strict rule that food and scientific samples should not be mixed, for obvious reasons. There are therefore separate refrigerators for food and samples, with large signs indicating the different uses. In a smaller lab, refrigerators can be divided between food and samples, with large signs stating “food” or “no food.” However, for the scientist who regularly works at this lab, there is no real reason to post such signs, as the person working there knows which refrigerator is which.

In small-team laboratories, space is often divided between lab machines, each run by one individual. In one such laboratory, there were nine people, each responsible for his or her own machine. Food and drink were not allowed in the lab: “This is for our own safety,” one of the scientists explained. The scientists wore lab coats and gloves when working at the machines; however, they removed them when sitting or when working at computers to avoid getting chemicals on the chairs and computers. When asked what they did when a colleague broke a rule, they said that they would remind the person directly. In the lab section, lab coats and gloves were worn as a matter of routine, no matter whether or not the experiment involved toxic substances. As the people working in the lab knew one another, it was easy for them to remind one another if someone forgot to follow the safety protocol. There was no need for external enforcement of the rules, as lab users monitored one another.

In the large laboratories, exemplified by the cleanroom, dust particles can destroy experiments on the nanoscale, and all kinds of contaminating factors must therefore be filtered out and controlled. In cleanrooms, scientists book machine time and may work beside total strangers. To enter the lab, one first must take a mandatory safety course and, officially, no one without security clearance is allowed to enter. Before entering the actual laboratory, the scientists must put on cleanroom suits to cover the entire body except for the face. The purpose of the suit is not to protect the scientists from hazardous chemicals but to protect the experiments from humans. Humans, on average, shed about 10,000 flakes of skin per minute, each potentially threatening a nanoscale experiment.

Inside the laboratory, typically several people are working at the same time and many machines are in great demand. In the cleanroom, as one works beside strangers wearing suits, it is difficult to recognize people. To keep to the schedule and maintain safe conditions, the following of protocol is essential. Inside the cleanroom, there is a system for ensuring that everyone follows protocol (Fig. 1). There are engineers to report to if scientists observe any violation of protocol and there are cameras inside the laboratory to monitor and record behavior. People inside the cleanroom are also asked to report violations of protocol to the cleanroom engineers. As the cleanroom is a shared space, it is essential that everyone there follow protocol. Breaches of protocol may put not only oneself at risk but others as well, which means that official control is required. The cleanroom engineers may issue official reprimands and even expel scientists from the cleanroom.

These ethnographic observations of safety protocols for laboratory work suggest that different laboratories produce different risk behaviors [15, 16]. From a social perspective, three ideal types of laboratories can be identified. First, there are single-person laboratories in practice used by just one individual. Second, there are the small-team laboratories in which the same two to ten people share one laboratory space on a daily basis but conduct their research individually. Third, there are large shared laboratories where changing selections of people conduct specific experiments. Machine time must be booked in advance in such laboratories, and the number of researchers passing through might be large. The rigor of protocols seems to increase with the number of scientists working in the lab, and the fewer the scientists in the lab, the fewer the regulations. Both upstream and downstream scientists use all three types of laboratories; at some stage, upstream scientists will need a cleanroom laboratory, which is available in large facilities, while toxicologists who experiment on living matter tend to use individual or small-team laboratories. So, if we look at laboratory risk management practices, there is no discernable divide between the two groups of nanoscientists. The divide becomes apparent first when they are asked to talk about risk.

## Perception of Risk Among Upstream Nanoscientists

The upstream nanoscientists at both field sites in Santa Barbara and Gothenburg were mostly electrical or physical engineers. The vast majority of them were experimentalists, meaning that they spent most of their workdays in laboratories. Most of these nanoscientists did not embrace the understanding that nanomaterials were a potential hazard. Risk associated with nanomaterials was not discussed and did not seem to bother them. Risk was not an issue. Emily York [28], who has conducted ethnographic fieldwork among nanoengineers, notes that, during their education, nanotoxicology is introduced as a separate field of science. She concludes that nanoengineers therefore regard the risk assessment of nanoparticles as part of a separate discipline, not part of nanoengineering.

The first time the first author heard a scientist proposed that nanomaterials might be dangerous was in June 2004. As a PhD student, the first author was invited to a conference arranged by the Swedish Research Council, titled “Nanotechnologies: Problems and Possibilities.” The purpose of the conference was to encourage scientists working on nanotechnology to begin to consider ethics. A scientist at the conference said that carbon nanotubes, i.e., cylindrical carbon molecules, might be dangerous as they are similar in structure to asbestos. In those days, there were no standards requiring the wearing of lab coats and gloves in the smaller labs. Nanoscientists producing carbon nanotubes in the laboratory did not wear protective wear. The potential hazards of nanomaterials were not discussed and this issue was not a concern among the nanoscientists. A typical response from a nanoscientist asked about risks and hazards at work was: “I really don’t know what the dangers are—most things are dangerous, for example, sun tanning and drinking can kill you.” Another response was, “I don’t worry about risks at all. I trust the administration to take care of that. It is really well organized: some make mistakes but the safety system is good. I’m relaxed and not afraid.”

An interesting observation during fieldwork among upstream nanoscientists working in the laboratory is that many had emotionally charged, almost familiar, and close relationships with the materials they worked with. For example, a nanoscientist working on carbon nanotubes offered the following passionate statement:

Nanotubes are like clothes—they are a material, but it is a very expensive material. It is like salt in food, it makes it tastier. Nanotubes form a sexy material that is sturdy and light. Nanotubes are like a very nice dress in the fashion world. Everyone wants to own that dress. It is a hot topic … I like to be around hot topics. I want to work with new stuff. I’m curious about how they behave [i.e. the nanotubes]. Every day there is something new. Today they are curly, tomorrow they are straight. If they become different the next time they are grown, it is not because of them, it depends on the temperature, pressure, etc. I think we have to let them grow as they like, we cannot control them today. We have to accept their behavior. We have to give them a little freedom.

Working with nanomaterials on a daily basis makes the upstream nanoscientists familiar with the material. A personal relationship is construed to the material, which become “friendly” and familiar. There is personal engagement with the material which was even described as “living matter” as explained to me by one nanoscientist “They [i.e. nanomaterials] are like babies, you need to educate them”.

However, there is a general understanding among upstream nanoscientists that working with chemicals can be dangerous. It is therefore important for the nanoscientists that they monitor themselves and others to ensure proper laboratory behavior in order to avoid accidents and risk exposure. This is done as we have seen by following protocols, which means that the laboratories have strict regulations for how to proceed safely with experiments. Following proper procedures was described by one nanoscientist as “the knowledge that you understand what is happening, that puts you at ease. You know how to deal with the chemicals you are using.” Nanoparticles, however, is not seen as more threatening than other materials and thus the protocols used are for larger scale compounds. Accidents are often seen as caused by the faulty behavior of the scientists themselves. This is also the reason why many laboratory accidents are not reported, as they are seen as resulting from human error.

During the first author’s fieldwork in Santa Barbara, a cartoon strip went viral among the nanoscientists. The same cartoon also appeared among the nanoscientists at Chalmers. It depicts two individuals in a restaurant. The first person, the scientist, orders a glass of H_2_0, i.e., water, while the second person tells the waiter “I want some H_2_0 too.” The waiter returns with a glass of H_2_0 (water) and a glass of H_2_0_2_ (hydrogen peroxide). The person ordering the H_2_0_2_ dies, as hydrogen peroxide is a toxic substance commonly used for hair bleaching. The scientist in the cartoon even calls the dead person an idiot for not knowing the difference between the two substances. The cartoon illustrates the strong norm of proper knowledge inside the group and how lack of knowledge can be lethal.

## Perception of Risk Among Downstream Nanoscientists

Toxicology is the science field for the study of harmful effects of chemical or physical compounds on biological systems [55]. The field is transdisciplinary and includes disciplines such as physics, biology, engineering, and chemistry. The sub-discipline of particle toxicology developed from research on the ill-health of miners related to lung disease and asbestos [56]. For particle toxicologists, the study of nanoparticles is nothing new, as the study of ultrafine materials has been going on for decades. “Ultrafine materials” have been rebranded as “nanoparticles” ([2, 55]: 155, [57]).

Since toxicologists study hazardous substances, their focus is not on the nanomaterial itself but on its risks. One toxicologist explained that “Nanotechnology may be the breakthrough technology for sustainable technology, but it also has risks,” and “New technology is very important for many people. Lots of patents in nano, but it is important to mention the risk of nanoparticles.” Corroborating Powell’s findings (2007), in contrast to upstream nanoscientists, among downstream nanoscientists, risk is a salient topic. One toxicologist told that toxicologists provide information about nanoparticles, but that it is up to society to decide what to do with that information:

The studies we do are important, but do all researchers working on cancer think they will cure cancer? Not so. I just provide information, and then the public must decide. This is your job, Mikael, to inform the public. We don’t know yet, but there seems to be some bad stuff.

The toxicologists, perceive the risks posed by nanomaterials as concrete and directly experienced: they actually witness cell death caused by nanoparticles in their laboratory environment. The toxicologists also view risk as complex, since different types of nanoparticles have different properties and behaviors. For example, powder forms are potentially more dangerous than liquids, and smaller particles are potentially more dangerous than larger ones.

The first thing learnt from engaging with the toxicologists in their laboratory environment working on nanoparticles, is that the potential risk of nanoparticles involves many confusing parameters. A professor explained:

There are different risks for different nanotechnologies. The exposure is not the same from socks as from tennis rackets. It is important where the exposure occurs, where the anticipated harm could occur … This is important for toxicology: even if the mass of the particles is the same, the size of the particles matters. How much surface area do the particles have? The shape of the particle is also important. Some shapes cause harm. Some nanomaterials do travel from the nose to brain in rats. If they [i.e. the particles] were larger, they would not have been able to travel to the brain. Different sizes produce different responses in rats, even if the particle mass is the same. The problem is that we do not know why.

Different risks are associated with different nanomaterials, and the exposure of an organism to a substance is crucial. The size and shape of the particles influence their toxicity; some shapes are potentially more harmful than others. This diversity of risk parameters is of course a huge difficulty when it comes to explaining the potential dangers of nanomaterials to the public, which is unaware of the complexity and the uncertainty. The downstream nanoscientists believed that the public want simple answers. Is the material safe? Yes or no? Lay people, according to the toxicologists, tend to lump all kinds of materials together, which does not make sense to the toxicologists. The only question that really seemed to anger some toxicologists was the, to them, naïve question “Are nanomaterials dangerous?” They could not answer this question, as there are so many different kinds of nanomaterials, some dangerous, some neutral, and some even beneficial to cell growth. From the lay perspective, harmful materials are dangerous in themselves, while the toxicologists claim that the dosage, shape, and size of the particles are what create the poison.

With so many parameters to understand, the toxicologists have to deal with numerous uncertainties when it comes to exposing themselves to harm from nanoparticles in the laboratory. Regarding his own safety, a toxicologist working daily with nanoparticles described the situation as follows:

We don’t know about the risk of nanoparticles. We think of the samples as risky and we are thinking about buying respirators. Other than that, it is just standard precautionary practice, as we are dealing with potentially toxic substances. I’m not afraid. As a scientist, you cannot work if you are afraid. This is our job. We work with fume hoods, protective gloves, glasses, lab coats.

Similar stories were repeated to me on several occasions:

Some particles are more risky than others, but we can’t say whether nano [as a whole] is risky or not. In two to three years, we may know more about what particles are dangerous or not. Metal particles together with salt seem to be more dangerous. Carbon nanotubes are like fibers and puncture cells, but if encapsulated they’re okay.

Toxicologists deal with unknown, potential harmful nanoparticles by following standard safety protocols, essentially the same procedures that upstream nanoscientists use to manage the risk of exposing themselves to nanoparticles. The protocols used, however, are designed for larger particles, meaning that nanoscientists and toxicologists wear lab coats and gloves and use fume hoods when dealing with powders. The belief in following protocol to avoid danger is fundamental.

The toxicologists are not as emotionally attached to nanomaterials as the upstream scientists who study and work with the materials themselves, as it is their job to study the adverse effects of nanomaterials. They are therefore more likely to treat nanomaterials as potentially toxic. The perceived risk of nanoparticles is also visualized by toxicologists. Once, during fieldwork, the first author was sitting next to a toxicologist using a scanning electron microscope to examine nanoclay, i.e., nanoparticles of layered mineral silicates. Such microscopes use electrons to create images on the nanometer scale. The toxicologist told me that nanoclays are used everywhere, in paint, toothpaste, cosmetics, and other consumer products: “You don’t expect a handful of dirt to be dangerous.” The computer screen showed an image consisting of a landscape of sharp needles. The toxicologist continued to explain:

This is the reason why I’m using a mask and fume box today. Really nasty! Did not use them in the beginning. This looks even nastier then those found before. The individual needle is about 80 nm wide. Here I do not need to see the whole landscape, the needles are enough. Looking at this under a microscope always scares me. It’s easy to miss the needles unless you go down really small. It [i.e. the landscape] looks nice and uniform, but if you look at it really closely, it is needles.

Here, we have a toxicologist who assesses a risk from seeing how bad the needles look. Toxicologists, in contrast to upstream nanoscientists, experience risk posed by nanomaterials in their daily work practice in the laboratory.

## Discussion

Laboratories can be understood as landscapes that shape scientific beliefs among its inhabitants. As early as 1934, Jakob von Uexküll explored the notion of Umwelt. This term focuses on the symbiosis between an experienced self-world and its surroundings. Different beings experience different self-worlds even though they share the same environment, so different types of scientists have different Umwelts ([58]: 76–78). Astronomers gaze with the aid of optical instruments at the universe, while chemists try to understand how the elements constitute substances. Each field of science, according to von Uexküll, explores a tiny sector of nature. This means that even though both upstream and downstream nanoscientists work in the same or similar laboratory environments, they occupy different Umwelts and accordingly perceive potential risks of nanoparticles in different ways. Tim Ingold [59], drawing on the Umwelt concept, uses the term “taskscape” to describe how technical practices are embedded in sociality and landscape. Tasks, in this setting, are activities carried out by skilled agents in an everyday life environment ([59]: 158). The taskscape is a socially constructed landscape of human activity that is under constant change and reinterpretation. Although laboratories are stable in their basic structure, they also change with new machines arriving and people coming and going. A term similar to taskscape has been introduced by Arjun Appadurai [60], who uses “technoscape” to refer to transnational flows of technologies, a phenomenon that constitutes an important part of the nanoscientists’ and toxicologists’ laboratory environment.

By interacting with nanoparticles on a daily basis, nanoscientists and toxicologists create understandings of nanoparticles as risky or safe, based on personal experience arising from their Umwelt/taskscape. It is interaction with nanomaterials in the experienced self-world situated in the lab environment that reinforces or changes researchers’ views of risk. The views created through this daily interaction with nanomaterials and laboratory colleagues are enforced when scientists write academic papers for specialized audiences, as “scientists tend to identify with particular fields or subfields in their writings as they tie their work to previous studies…” ([14]: 427). Both nanoscientists and toxicologists not only work in separate laboratories and write in separate journals, they also travel globally between separate meetings and institutional setups, each comprising a limited number of connected research facilities, in what can be called intra-space mobility ([61]: 514). This intra-space mobility restricts the range of interaction between upstream and downstream nanoscientists and thereby the exposure to external influence in terms of other view points and perspectives.

This article has through ethnographic lens contributed to an enhanced perspective on how nanoscientists perceive risk related to nanoparticles through an “inside perspective” and how this view is sustained by their respective *Umwelt.* Nanomaterials are interlocked with technology in laboratory landscapes where science work is practiced. The nanoscientists share the idea that laboratory work is potentially dangerous, that it should be safe and that strict protocols for laboratory conduct contribute to work place safety. In addition, they also believe that protocols are part of sound science procedures in that they contribute to standardized and “clean” laboratory environments necessary for reliable experimental studies. So far, upstream and downstream nanoscientists are in agreement about risk management of nanomaterials. When it comes to beliefs and attitudes about nanomaterials, more broadly, the two categories of scientists differ. Upstream scientists emphasize the innovative potential of the new materials, which they think can be beneficial to society; they express an affectionate and personalized stance towards the material; they emphasize their control of the material and speak about how it is refined and developed; and they downplay risk as a topic of relevance. The downstream scientists present a distinctly different narrative. They focus on the high uncertainty and unpredictability of nanomaterials; the materials are portrayed as unstable, difficult to assess, and potentially very dangerous; and they are concerned about how toxicological findings can actually be communicated to the general public, who they assume, want only simple answers.

In agreement with previous research on upstream and downstream scientists and disciplinary culture, the results of this study suggest that the expert science role needs to be approached with caution. There is no unitary expert science perspective on risk with nanotechnology. Indeed, there are many expert perspectives and states of affect vis-à-vis nanotechnology [62] built from disciplinary background, science culture, laboratory practices, and social interactions within organizational and institutional research networks and infrastructures (specialist conferences, research collaborations, laboratory facilities, and science departments). In the broader discussion of nanotechnology in society, its role and contribution, ethical issues, and regulatory issues, many different scientific experts need to be involved.
